# Effect of microbial cell preparation on renal profile and liver function among type 2 diabetics: a randomized controlled trial

**DOI:** 10.1186/s12906-015-0952-5

**Published:** 2015-12-12

**Authors:** Somayyeh Firouzi, Barakatun-Nisak Mohd-Yusof, Hazreen-Abd Majid, Amin Ismail, Nor-Azmi Kamaruddin

**Affiliations:** Department of Nutrition and Dietetics, Faculty of Medicine and Health Sciences, University Putra Malaysia, 43400 Serdang, Selangor Malaysia; Research Centre of Excellence for NCD (Nutrition and Non-communicable Diseases), Universiti Putra Malaysia,, 43400 UPM Serdang, Selangor Malaysia; Centre for Population Health and Department of Social and Preventive Medicine, Faculty of Medicine, University of Malaya, Kuala Lumpur, Malaysia; Department of Medicine, Faculty of Medicine, University Kebangsaan Malaysia, Kuala Lumpur, Malaysia

**Keywords:** Microbial cell preparation, Probiotics, Urea, Renal profile, Liver function tests, Type 2 diabetes, *Lactobacillus*, *Bifidobacterium*

## Abstract

**Background:**

The beneficial effect of probiotics on renal profile and liver function has been reported among patients with chronic kidney disease and fatty liver respectively. However, its effect on renal profile and liver function among type 2 diabetic individuals has not been fully understood. To investigate the effect of microbial cell preparation on renal profile and liver function tests among type 2 diabetic individuals.

**Methods:**

A randomized, double-blind, parallel-group, controlled clinical trial was conducted on a total of 136 type 2 diabetics age 30-70 years old in a teaching hospital in Kuala Lumpur, Malaysia. Subjects were randomly assigned to receive microbial cell preparation (*N* = 68) or a placebo (*N* = 68) for 12 weeks. The outcomes measured at baseline, week 6, and week 12 and included changes in renal profile (Sodium, Potassium, Urea, Creatinine, Glomerular Filtration Rate), and liver function tests (Albumin, Total Protein, Alkaline Phosphatase, Alanine Aminotransferase, Aspartate Aminotransferase). Intention to treat (ITT) analysis was performed on all the recruited subjects, while per protocol (PP) analysis was conducted on those who completed the trial with good compliance.

**Result:**

The urea levels significantly declined in the probiotic group. Serum urea levels reduced from 4.26 mmol/L to 4.04 mmol/L in Probiotic Group while it increased in Placebo Group from 4.03 mmol/L to 4.24 mmol/L. These changes were significant between groups in ITT analysis (*p* = 0.018). Other parameters did not change significantly between groups.

**Conclusion:**

12 weeks supplementation with daily dosage of 6 × 10^10^ Colony Forming Units of multi-strain microbial cell preparation significantly improved urea levels.

**Trial registration:**

(Clinical trials: #NCT01752803)

**Electronic supplementary material:**

The online version of this article (doi:10.1186/s12906-015-0952-5) contains supplementary material, which is available to authorized users.

## Background

Type 2 diabetes individuals are at risk of chronic kidney [1] and liver diseases [2]. Recent literature proposed that modulation of gut microbiota with probiotics have the potential to improve renal profile and liver function. Proof for the above purport is that, gut microbiota is altered in patients with Chronic Kidney Disease (CKD) [3] and non-alcoholic steatohepatitis [4] as compared with healthy subjects. Different small-scaled clinical trials in hemodialysis patients [5, 6], or stage 3 and 4 of CKD [7, 8], reported a non-significant improvement on different indices of renal profiles following a period of probiotic consumption ranging from 2 to 6 months. In terms of liver function, a meta-analysis of four randomized controlled trials, comprising of 134 non-alcoholic fatty liver and non-alcoholic steatohepatitis patients, demonstrated significant improvements in well-recognized clinical markers of liver damage including Alanine Aminotransferase (ALT), Aspartate Transaminase (AST) and Tumor Necrosis Factor Alpha (TNFα) [9].

Liver damage is partly attributed to the increased systematic inflammation by trans-locating gut harmful bacteria into the blood stream. Probiotics has a beneficial role in liver diseases through diminishing proliferation of harmful bacteria in the gut and improving the integrity of gut mucosa [10]. Indeed, gut microbiota is altered in CKD. The reason might be due to uremia which impairs intestinal barrier function and promotes inflammation throughout the gastrointestinal tract. This alteration may interfere with the normal functions of the gut, causing disturbances in renal parameters. Therefore, manipulation of gut microbiota may positively impact renal profiles [3].

Despite promising findings from the above studies, it is still uncertain whether data from hemodialysis, CKD patients or non-alcoholic fatty liver and non-alcoholic steatohepatitis patients can be extrapolated to type 2 diabetics who are at risk of CKD or chronic liver diseases. Therefore, this randomized controlled trial is aimed to investigate the effect of a multi-strain microbial cell preparation on renal profile and liver function among type 2 diabetic individuals.

## Methods

### Subject selection

This study was a double blind randomized parallel-group, placebo controlled trial which was conducted at the diabetes clinic of a teaching hospital in Kuala Lumpur, Malaysia. Type 2 diabetic individuals with stabilized dose of medication, age 30 to 70, with Glycated Hemoglobin A1c (HbA1c) between 6 and 12 %, Fasting Blood Glucose (FBG) <15 mmol/l and Body Mass Index (BMI) between 18.5 and 40 kg/m^2^, were eligible to join the study (the levels of BMI and HbA1c extended from the initial plan due to the difficulties fulfilling the sample size). Exclusion criteria include subjects who were treated with insulin, antibiotics and/or other medication which might interfere with the results of the study and having any acute or chronic disease other than diabetes, hyperlipidemia, and hypertension.

All the patients attending the diabetes clinic from February 2012 to December 2013, comprising 6967 subjects, were screened for the study. A total number of 456 eligible subjects, whose contact numbers were available at the hospital system, were approached by phone contact. During the conversation, the benefits and risk of the study was briefly explained to the eligible subjects. Those who were willing to join the study had an appointment with the main researcher to describe the details of the study and to double check inclusion and exclusion criteria in a face to face interview. Subjects were given adequate time to consult with their doctors and families and in order to stop taking any probiotic or prebiotic source. Among them, 136 subjects agreed to join the study and were randomly allocated to receive either probiotic (refers to microbial cell preparation) or placebo for a 12-week period with the allocation ratio of one. The subjects signed the consent form before recruitment. The study protocol was approved by the Clinical Research and Ethics Committee of Universiti Kebangsaan Malaysia Medical Center according to the regulations of declaration of Helsinki. The protocol of the study was registered at the U.S. National Institute of Health website (http://www.clinicaltrials.gov) #NCT01752803. Data collection finished on May 2015.

A 0.6 mmol/L difference in urea level with 1.1 mmol/L standard deviation yields a significant difference with a power of 80 % [7]. Using Lemeshow et al. equation [11], the number of subjects per group became 57, and considering a 20 % drop out rate, this number reached 68 subjects per group.

### Intervention procedure

The random allocation sequence was generated by the main researcher using a computer model (accessible through http://www.randomization.com website) with blocks of four and eight in order to allow having exact number of 68 in each group. Both groups received standard Medical Nutrition Therapy (MNT), aimed to homogenize the food intake of the subjects regardless of their assigned group. The amount of energy was calculated using the quick method formula [12]. The proportion of macronutrients was according to the medical nutrition therapy for type 2 diabetes mellitus [13]. Subjects were asked not to change their levels of physical activity and do not take any source of probiotic during the course of the study. The measurements were conducted at baseline and week 12 with at an interim analysis at week 6. The main researcher was in charge of recruitment, allocation, data collection, educating MNT and statistical analysis.

### Blinding

The probiotic and placebo sachets were identical in weight, appearance, texture, nutritional value (except having probiotic in probiotic sachets), as well as smell. They were only recognizable by a code on them (A or B). The researchers and the subjects were blinded to the content of the sachets throughout the study and during the statistical analysis. After conducting statistical analysis, the database was locked and the codes were revealed to the researchers at the present of a representative from the company and a witness from the department.

### Adverse effects

The incidence of adverse effects was checked at each visit and by additional phone calls between each visit. Subjects were asked to report any symptom that occurred after commencing probiotic consumption or any changes in the severity of their ongoing conditions. The expected adverse effects were minor gastrointestinal disturbances. Subjects who had severe gastrointestinal disturbances or any other adverse effects were asked to stop the supplement and referred to the physician. Those who stopped taking the supplement due to the adverse effects were requested to continue the trial in order to enter the respective data into the Intention To Treat (ITT) analysis.

### Supplementation and compliance

Microbial cell preparation composed of six viable freeze dried microorganism strains including *Lactobacillus acidophilus*, *Lactobacillus casei*, *Lactobacillus lactis*, *Bifidobacterium bifidum*, *Bifidobacterium longum* and *Bifidobacterium infantis* with a daily dose of 6 × 10^10^ (Hexbio® B-Crobes Laboratory Sdn Bhd. Ipoh; Malaysia). Sachets were kept at a dry place below 25 °C away from direct sunlight and subjects were also asked to do so. The shelf life of sachets was 2 years and the sachets were delivered to the study site at 4-month intervals. Subjects were asked to pour the contents of the sachets in one glass of water (approximately 250 ml) and drink twice per day (morning and evening) with or without a meal. Subjects were asked to bring their remaining sachets at each follow up visit. Sachet count method was used to determine subject compliance, with a rate of 85–100 % considered acceptable [14].

### Measurements of outcomes

Primary outcome measures were parameters of renal profile and liver function tests. Secondary measurements including anthropometry, BMI, Energy, macronutrient and micronutrient intake as well as physical activity and sedentary behavior were also assessed in order to ensure the comparability between two groups in terms of baseline characteristics and changes throughout the study. The measurements were done at baseline, week 6 and week 12.

The weight was taken using a digital weighing scale (SECA; London British Indicators, UK). Height was measured only at baseline with a height attachment on the same weighing scale (SECA; London British Indicators Ltd). Waist Circumference was measured with a flexible tape. All anthropometric measurements were done twice to the nearest one decimal and the average of the measurements was considered as the final figure. BMI was then calculated. Subjects were categorized as normal weight or Overweight and Obese (OW/OB) according to the WHO classification [15].

Energy, macronutrient and micronutrient intake were measured by 3-Day Diet Records (3DDR) from each visit. Nutrient analysis was performed using a computerized dietary analysis program (Nutritionist Pro Version 2.0; First Data Bank, The Hearst Corp., New York). Physical activity level was assessed using a short version of the International Physical Activity Questionnaire (IPAQ) [16].

Blood samples were drawn by trained laboratory technicians through the antecubital arm vein and analyzed immediately. Sodium and potassium levels were detected through ion selective electrode ion dissolved assay. Urea levels were measured by urease method. Creatinine levels were detected by Jaffe method. Total protein levels were determined by Biuret reaction. Albumin levels were detected through Bromocresol Green Dying method. Total bilirubin was measured by a 2, 4-dichlorophenyl diazonium method. The levels of ALT and AST were detected using a modified method of the International Federation of Clinical Chemistry. The levels of Alkaline Phosphatase (ALP) were detected through Adenosine Mono Phosphate buffer. All the measurements were done using the Cobas® 8000 modular analyzer, series 702 (Roche Diagnostics, Mannheim, Germany). The expected normal values were 135–150 mmol/L for sodium, 3.5 to 5 mmol/L for potassium, 2.5–6.4 mmol/L for urea, and 44 to 80 μmol/L for creatinine. Total protein: 67–88 g/L, albumin: 3.5 to 5 g/L, bilirubin: <23 μmol/L, ALT :< 44U/L, ALP: 32–104 U/L, and AST: >45 U/L.

Glomerular Filtration Rate (GFR) was calculated using equation 7, developed by the modification of diet in renal disease study [17]: GFR = 170 × [serum creatinine concentration (mg/dl)]^-0.999^ × age ^-0.176^ × [serum urea nitrogen concentration (mg/dl)^-0.17^] × [albumin concentration (g/dl)^-0.318^ × (0.762 if the patient is female) × (1.18 if the patient is black).

### Assessment of probiotics recovery in stool

Recovery of the supplement in stool was assessed by quantification of *Lactobacillus* and *Bifidobacterium* species at baseline and week 12 on a subsample of 40 subjects. The Colony Forming Units (CFUs) of *Lactobacillus* and *Bifidobacterium* species were counted by plate counting method. The accuracy of counted colonies was confirmed through Polymerase Chain Reaction (PCR) (for the details of the procedure see Additional file 1).

### Statistical analysis

Statistical analyses were performed using SPSS version 22 (SPSS Inc. Chicago, USA). The differences between tests were considered significant if the two-tailed *p* values was <0.05. Normality was confirmed by Shapiro-wilk test. Log transformation was used to obtain normal data from skewed variables. The variables are reported as mean and Standard Deviation (SD) as well as 95 % confidence interval. Independent sample *t* test and Man Whitney *U* test as well as Chi-square test were used to determine the differences between groups at baseline.

ITT analysis was conducted on the full set of data with imputing method where needed. Last observation carried forward was used for imputing missing data. Per Protocol (PP) analysis was conducted on those subjects who successfully finished the third visit with more than 85 % compliance. The changes in variables over the course of the study were analyzed - within each group and between groups - by General Linear Model Analysis of Variance (GLM ANOVA). In this study, the results from the ITT analysis are presented in the tables and graphs, while the PP analysis results are demonstrated by *p* values only. Effect size was detected for variables with significant improvements between groups, and classified as: 0.01–0.05 considered small, 0.06–0.13 medium, and ≥0.14 considered large [18].

## Results

### Socio-demographic characteristics and compliance rate

A number of 136 type 2 diabetic subjects (52.2 % male) -with mean age of 53.5 ± 8.5- agreed to join the study and were randomly allocated to probiotic (*n* = 68) or placebo (*n* = 68) groups. The mean compliance rate was 86.1 % in the Placebo Group and 89.0 % in the Probiotic Group, which was comparable between groups (*p* = 0.319). The two groups were comparable at baseline in terms of age, BMI, dietary intake, physical activity level (Table 1), treatment modalities and co-morbidities (Table 2), renal profile and liver function tests (except for ALT and AST) (Table 1).

**Table 1:** Baseline data of subjects in each group

Baseline parameters	Probiotic group (*n* = 68)	Placebo group (*n* = 68)	*P* value
Age	52.9 ± 9.2	54.2 ± 8.3	0.362
Gender (male)	37 (54.4 %)	34 (50 %)	0.731
Ethnicity			
• Malay	31 (45.6 %)	38 (55.8 %)	0.601
• Chinese	18 (26.4 %)	17 (25 %)	
• Indian	16 (23.6 %)	11 (16.2 %)	
• Other races	3 (4.4 %)	2 (3 %)	
HbA1c	7.6 ± 1.3	7.5 ± 1.3	0.795†
Physical characteristics			
• Weight (kg)	74.6 ± 15.1	76.6 ± 15.6	0.514
• Height (cm)	160.0 ± 8.4	161.8 ± 9.4	0.285
• BMI (kg/m^2^)	29.2 ± 5.6	29.3 ± 5.3	0.837
• WC			
Male (cm)^a^	100.4 ± 13.5	102.0 ± 13.7	0.429
Female (cm)^b^	97.3 ± 14.6	96.7 ± 9.5	0.618
Diet intake			
• Energy (kcal)	1473 ± 402	1508 ± 503	
• % of calorie by carbohydrate	54.1 ± 7.9	53.9 ± 8.2	0.917
• % of calorie by protein	16.3 ± 3.9	17.1 ± 3.7	0.612
• % of calories by fat	29 ± 2	28.6 ± 5.4	0.917
• Fibre (gr)	7 ± 5	6 ± 4	0.283†
• Sodium (gr)	1522 ± 639	1840 ± 919	0.065
Physical activity level			
• Total physical activity score (MET_min/wk)	1784 ± 2100	1989 ± 1869	0.570
• Sedentary activity (hours/day)	6.2 ± 3.3	5.5 ± 3.0	0.212
Renal profile			
Sodium (mmol/L)	138.5 ± 2.2	137.9 ± 2.5	0.094
Potassium (mmol/L)	4.42 ± 0.30	4.40 ± 0.40	0.284
Urea (mmol/L)	4.26 ± 1.29	4.03 ± 0.89	0.069†
Creatinine (μmol/L)	69.20 ± 17.36	72.10 ± 18.84	0.326
GFR (ml/min)	74.45 ± 18.5	73.66 ± 13.38	0.423
Liver function tests			
Albumin (g/L)	45.64 ± 3.22	45.51 ± 2.53	0.713
Total protein (g/L)	74.24 ± 4.93	73.87 ± 3.73	0.780
Bilirubin (μmol/L)	9.77 ± 3.50	10.38 ± 3.92	0.417†
ALT (U/L)	23.20 ± 9.65	32.53 ± 16.10	0.001*†
ALP (U/L)	68.49 ± 23.19	73.42 ± 18.11	0.088
AST (U/L)	20.1 ± 4.7	25.8 ± 7.1	0.002*

Results are expressed as Mean ± SD or *n*(%) where appropriate

*Abbreviation*: OAD: Oral Anti-Diabetic Agent, OW/OB: Overweight and obese, HbA1c: Hemoglobin A1c

† log transformed independent sample *t*-test

*significant difference between two groups

^a^ *n* = 34 in Placebo Group, *n* = 31 in Probiotic Group, ^b^ *n* = 34 in Placebo Group, *n* = 37 in Probiotic Group

**Table 2:** Treatment modalities at baseline

Baseline parameters	Probiotic group (*n* = 68)	Placebo group (*n* = 68)	*P* value
	n (%)	n (%)	
Diet alone	6 (8.8 %)	1 (1.4 %)	0.660
Single OAD	19 (28.0 %)	16 (23.6 %)	0.589
Dual OAD	37 (54.4 %)	40 (58.8 %)	0.912
>2 types OAD	6 (8.8 %)	11 (16.2 %)	0.486
On Antihypertensive	47 (69.2 %)	48 (70.6 %)	0.943
On Anti-hyperlipidemic	54 (79.4 %)	58 (85.4 %)	0.610
Obesity	18 (26.5 %)	14 (20.6 %)	0.419

### Attrition rate and incidence of adverse effects

**Fig. 1:**
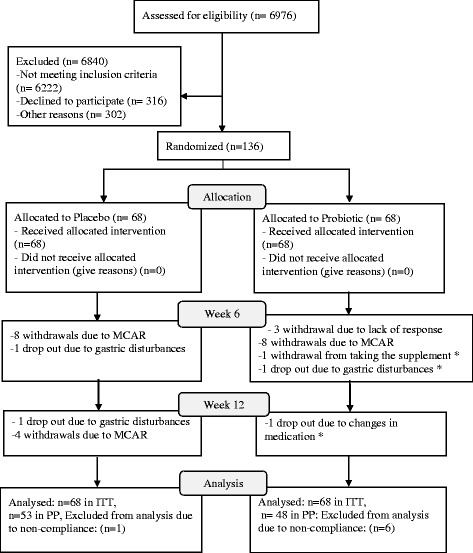
CONSORT flowchart

The attrition rate was 20.6 % at the end of 12 weeks and with considering non-compliance this number reached 25.7 % (Fig. 1). Despite higher incidence of adverse effects in the Probiotic Group (8.7 %) compared with the Placebo Group (3.7 %), it was comparable between groups (*p* = 0.156). Most of the observed adverse effects were those of expected minor gastric disturbances. Besides, Carbuncle and sexual impotency each happened in one of the subjects from the Probiotic Group. Two subjects (1.5 %) in the Probiotic Group discontinued the probiotic supplement due to the adverse effects.

### Recovery of probiotics in stool

The *Lactobacillus* and *Bifidobacterium* species increased significantly in the probiotic group as compared to the placebo group (Refer to Additional file 2).

### Changes in dietary intake and physical activity levels over the course of study

The levels of physical activity, intake of energy and the percentage of energy derived from macronutrients as well as fiber intake did not change significantly over the course of the study between groups (Additional file 3). There was a significant change in the sodium intake between groups. A correlation analysis was carried out between sodium levels and all the parameters at each visit. No significant correlation was found between sodium levels and biochemical parameters, meaning that changes in sodium levels did not affect biochemical parameters. Thus, the subsequent analysis did not adjust for energy intake, percentage of macronutrients derived from energy, fiber, sodium intake as well as physical activity levels.

### Changes in renal profile parameters over the course of study

**Fig. 2:**
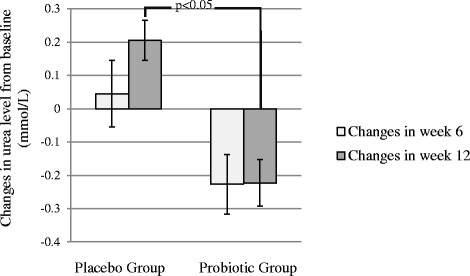
Mean changes in urea levels in Probiotic and Placebo Group over the course of the study

The levels of sodium and potassium did not change significantly within each group or between groups. The creatinine levels significantly decreased within each group while this decrease was not significant between groups. GFR, also, significantly decreased in the Placebo but the changes between the two groups were not statistically significant (Table 3). The levels of sodium, potassium, creatinine and GFR did not change significantly between groups in the sub-analysis of BMI categories. The changes in urea levels between groups were significant in the ITT analysis (small effect size of 0.031) but not PP (Table 3). This changes was significant from baseline to week 12 (*p* < 0.05) (Fig. 2).

**Table 3:** Renal profile and liver function tests throughout the study in Probiotic and Placebo Groups

	Probiotic Group (*n* = 68)			Within group *p* value	Placebo Group (*n* = 68)			Within group *p* value	Interaction *p* value	
	Mean ± SD				Mean ± SD					
	Baseline	Week 6	Week 12		Baseline	Week 6	Week 12		ITT	PP
Renal profile										
Sodium (mmol/L)†	138.5 ± 2.2	138.9 ± 2.7	138.1 ± 3.5	0.147	137.9 ± 2.5	138.8 ± 2.9	138.5 ± 3.1	0.167	0.235	0.280
Potassium (mmol/L)	4.42 ± 0.30	4.42 ± 0.31	4.35 ± 0.31	0.060	4.40 ± 0.40	4.34 ± 0.36	4.37 ± 0.43	0.360	0.164	0.351
Urea (mmol/L)	4.26 ± 1.29	4.03 ± 1.00	4.04 ± 1.04	0.086	4.03 ± 0.89	4.07 ± 1.10	4.24 ± 1.14	0.081	<0.05	0.181
Creatinine (μmol/L)	69.20 ± 17.36	70.87 ± 18.70	72.26 ± 19.73	<0.05	72.10 ± 18.84	71.95 ± 18.60	75.17 ± 18.93	<0.05	0.329	0.288
GFR (ml/min)	74.45 ± 18.5	74.14 ± 16.94	73.07 ± 17.13	0.710	73.66 ± 13.38	73.91 ± 13.58	68.89 ± 13.55	<0.05	0.147	0.197
Liver function tests										
Albumin (g/L)	45.64 ± 3.22	45.38 ± 3.18	45.48 ± 2.97	0.551	45.51 ± 2.53	45.81 ± 2.05	45.94 ± 1.93	0.339	0.147	0.224
Total protein (g/L)	74.24 ± 4.93	73.32 ± 5.14	73.03 ± 5.98	0.190	73.87 ± 3.73	72.33 ± 4.89	71.59 ± 5.19	<0.05	0.695	0.534
Bilirubin (μmol/L)†	9.77 ± 3.50	10.03 ± 3.59	10.09 ± 3.70	0.739	10.38 ± 3.92	10.27 ± 3.20	10.28 ± 3.20	0.996	0.260	0.845
ALT (U/L)†	23.20 ± 9.65	22.38 ± 10.13	22.33 ± 10.02	0.199	32.53 ± 16.10	32.59 ± 16.78	33.27 ± 17.29	0.823	0.421	0.374
ALP (U/L)	68.49 ± 23.19	68.28 ± 21.82	67.00 ± 21.77	0.209	73.42 ± 18.11	71.97 ± 18.57	71.29 ± 20.72	0.134	0.481	0.485
AST (U/L)	26.84 ± 77.12	24.58 ± 6.64	25.71 ± 6.81	0.441	20.12 ± 4.68	20.26 ± 5.12	21.29 ± 6.30	0.275	0.490	0.495

† log transformed GLM ANOVA

**Fig. 3:**
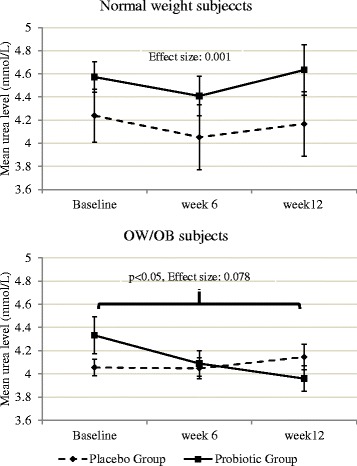
Mean urea levels between study groups in week 6 and week 12 among normal weight and OW/OB subjects

A sub-analysis was conducted in order to determine the effect of probiotic supplementation among normal weight and OW/OB individuals. The probiotic supplementation did not show any impact on urea levels among normal weight subjects, while it caused significant reduction in the urea levels in OW/OB subjects in Probiotic Group as compared to OW/OB subjects in the Placebo Group (only in ITT analysis). This difference was only significant between baseline and week 12 (*p* < 0.05) (Fig. 3).

**Fig. 4:**
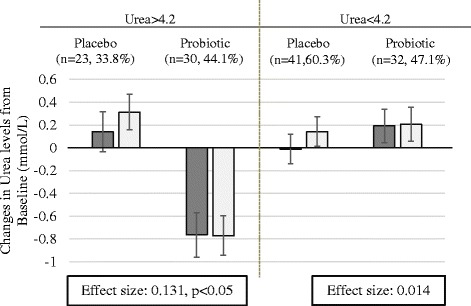
Comparison of mean changes in Urea levels between study groups according to their baseline value

Further analysis was aimed to demonstrate whether changes in urea concentrations in blood depend on the baseline urea levels. The average of the urea level at baseline (4.2 mmol/L) were used as cut off and the data split to those with lower levels of 4.2 mmol/L and those with higher levels of 4.2 mmol/L. Interestingly, in the group with urea levels of higher than 4.2 mmol/L, the changes in the urea levels between the Probiotic and Placebo Group was significant, while it did not show any significance in the other group (Fig. 4).

### Changes in liver function tests over the course of study

The mean of all the liver function tests did not change significantly between or within groups (Table 3). Except for total protein which was significantly reduced in the Placebo Group in within group analysis (*p* = 0.006).

## Discussion

### Changes in renal profile parameters over the course of study

Results of this study showed that probiotics have the potential to improve urea levels especially among OW/OB subjects and those with elevated levels of urea. However, other parameters of renal profile as well as liver function tests have not been affected by probiotics.

The mean levels of sodium and potassium in the blood have not changed after probiotic supplementation. The creatinine levels increased significantly within each group. This rise may be attributed to the intake of Angiotensin-Converting Enzyme inhibitor (ACE inhibitor) in the subjects of this study as previous literature had demonstrated that chronic ACE inhibitor administration increases the creatinine levels [19]. Surging levels of creatinine has led to subsequent significant decrease in GFR in the Placebo Group in this study. However, the mean creatinine levels did not show any changes between groups. This is in line with the study by Alatriste et al. [7], whereby eight weeks of supplementation with *L. casei shirota* did not contribute to any changes in creatinine levels in patients who were suffering from stage 3 and 4 of CKD.

In the current study, the ITT analysis found 5.2 % significant reduction in the urea levels in the Probiotic Group. In a study using subjects with stage 3 and 4 of chronic renal disease, a non-significant reduction of up to 3.4 % in urea level was noted after supplementation for two months with dose of 8 × 10^9^ *L. casei shirota* and a significant reduction of up to 11 % with the dosage of 16 × 10^9^ [7]. Despite higher dosages of up to 7.5-fold and 3.5-fold in the current study compared with 8 × 10^9^ and 16 × 10^9^ dosage used in the Alatriste et al. study [7], respectively; the reduction noted in our study was lower than the former. In addition, the duration of supplementation in the current study was four weeks longer than the Alatriste et al.’s [7] study. The reason for the higher decline in their study can be due to the higher baseline levels of urea in their study (approximately 4.53 mmol/L) compared with the present study (4.3 in Placebo Group and 4.26 in Probiotic Group). Similarly, this study showed that probiotic supplementation has better outcomes among those with high urea levels.

The amount of protein intake may influence urea levels [20]. However, the protein intake of the subjects in both group were comparable at baseline and did not change significantly over the course of the study. Thence, the changes in urea levels cannot be contribute to changes in protein intake. In the quest of linking probiotic supplementation with renal status, it is worth mentioning that patients with renal diseases usually have impaired intestinal microbiota. Majority of uremic patients have disturbances in gastrointestinal tract mucus and the intestinal ecosystem. Most of these patients have higher levels of aerobic bacteria such as *Escherichia coli (E.Coli)* and lower levels of anaerobic microorganisms such as *Lactobacillus* and *Bifidobacterium*. The increase in the levels of *E.coli* will contribute to a high production of urea and an increase in pH levels. By introducing lactic acid bacteria such as *Lactobacillus* and *Bifidobacterium*, the pH decreases through fermentation of carbohydrates [3, 7, 21]. Thence, these bacteria prevent proliferation of aerobic bacteria in the gut and promote a balanced microbiota in the gut [22]. Another possible explanation is degradation of urea by urease activity of selected probiotic species, e.g. *Bacteroides* [22].

Urea levels significantly improved after probiotic supplementation in OW/OB subjects. Yet, how changes in urea levels differ between normal weight and OW/OB cannot be explained easily. One possible theory may be related to the different proportion of the bacteria phyla found in OW/OB compared with normal weight subjects. Interestingly, obese people have higher proportions of *E. coli* [23]. Therefore, it can be hypothesised that probiotic supplementation induces more decline in the amount of *E. coli* in OW/OB subjects compared with normal weight subjects. This may partly explain why there is significant decrease in urea levels in OW/OB subjects compared with normal weight subjects. Overall, the results of the present study showed that probiotics are effective therapies for decreasing urea levels in OW/OB subjects.

### Changes in liver function tests over the course of study

In this study, the albumin and total protein levels were within the normal range at baseline and did not change significantly between groups over the course of the study. In within group comparison, total protein decreased in both groups while this decline was significant in the Placebo Group. The reason for within group changes remained unclear. Two human studies have shown some improvements in albumin and total protein levels after probiotic consumption among ulcerative colitis patients [24] and elderly hospitalised subjects [25]. However, these increases are contributed for protecting the integrity of the gut mucosa and overall well-being of the subjects due to the probiotics role in defecting infection, and thus cannot be generalized to diabetic individuals.

In the current study, no significant improvements were found with regard to other liver function parameters including bilirubin, ALT, ALP, and AST. The baseline values of ALP and AST were significantly higher in the placebo group. However, the GLM ANOVA automatically adjusts the analysis for baseline values. In terms of changes after supplementation, there is a growing body of evidence which shows improvements after probiotic supplementation in subjects with alcoholic [26–28] or non-alcoholic fatty liver disease [29, 30]. Overall, from the findings of this study it can be concluded that, probiotics are not effective in improving liver parameters among type 2 diabetic individuals with healthy liver status.

Despite the potential impact of probiotics on liver function, another aspect of determining liver function is predicting the safety of probiotic supplementation. Conventional liver function tests are determinants of drug-induced liver toxicity. The extent of liver injuries due to drug-induced liver toxicity varies from mild asymptomatic liver injuries to acute hepatocellular hepatitis which is detectable through changes in ALT [31]. In addition, each component of liver function tests represents a special function of the liver [32]. Therefore, not inducing any significant increase in these parameters in the current study indicates that the daily dose of 6 × 10^10^ CFUs of bacteria for three months have not contributed to any drug-induced liver injury among type 2 diabetic individuals.

The current study has several limitations which should be taken into account before generalization of the results. First of all, the recovery of the probiotic in the stool was tested among a subsample of the population. Although PCR method showed significant increase in the quantities of the *Lactobacillus* and *Bifidobacterium*, it would be more precise if 16S rRNA gene sequencing would have been used. Main researcher was in charge of randomization, while it would have had an added advantage if disclosing randomization sequence would have performed by a third person at the recruitment time. Indeed, stool analysis was conducted on a subsample of the subjects. The last is the small sample size and short duration of study. Larger sample size and longer duration of study would have been more appropriate to observe an improvement in the renal and liver function. Looking at the upside, this study is one of its kinds which assessed the impact of probiotic supplementation on healthy status of renal and liver among type 2 diabetes individuals who are at risk of renal failure or kidney injuries. Besides, in this study the subjects were matched in terms of diet intake and physical activity levels and had a high compliance to the supplement.

## Conclusion

Overall, this study showed that a multi-strain probiotic supplement consisting of *Lactobacillus* and *Bifidobacterium* with a dosage of 6 × 10^10^ it was beneficial in improving urea levels particularly among overweight and obese subjects and it did not induce any liver toxicity among type 2 diabetics. It is of utmost importance in the context of preventing chronic renal failure among type 2 diabetic individuals. Future research should target to demonstrate the mechanism behind this effect.

## Additional files

Additional file 1:
**Method of quantifying** ***Lactobacillus*** **spp. and** ***Bifidobacterium*** **spp.** (DOCX 133 kb) Additional file 2:
**Quantities of** ***Lactobacillus*** **spp. and** ***Bifidobacterium*** **spp. before and after intervention in both groups.** (XLSX 10 kb) Additional file 3:
**Changes in Dietary intake and physical activity level at baseline, week 6 and week 12.** (XLSX 11 kb)

## Abbreviations

CKD: Chronic Kidney Disease
ALT: Alanine Aminotransferase
AST: Aspartate Transaminase
TNFα: Tumor Necrosis Factor Alpha
HbA1c: Glycated Hemoglobin A1c
FBG: Fasting Blood Glucose
BMI: Body Mass Index
MNT: Medical Nutrition Therapy
ITT: Intention To Treat
OW/OB: Overweight/Obese
WHO: World Health Organization
3DDR: Three Day Diet Record
IPAQ: International Physical Activity Questionnaire
ALP: Alkaline Phosphatase
GFR: Glomerular Filtration Rate
CFUs: Colony Forming Units
PCR: Polymerase Chain Reaction
SD: Standard Deviation
PP: Per Protocol
GLM ANOVA: General Linear Model Analysis of Variance
ACE inhibitor: Angiotensin-Converting Enzyme inhibitors
*E. Coli*: *Escherichia Coli.*
