# Supplementary material for: Effect of microbial cell preparation on renal profile and liver function among type 2 diabetics: a randomized controlled trial
Source: BMC Complement Altern Med. 2015 Dec 12;15:433. doi: 10.1186/s12906-015-0952-5 (PMC4676823; doi:10.1186/s12906-015-0952-5)
Supplement: Additional file 1: — Method of quantifying Lactobacillus spp. and Bifidobacterium spp. (DOCX 133 kb) [file 12906_2015_952_MOESM1_ESM.docx]

**Phenotype identification**

The counted colonies were checked for colony morphology. The colony morphology was checked against the morphology of *Lactobacillus* and *Bifidobacterium* colonies obtained from the control plates.

*Bifidobacterium* colonies were transparent white circular raised and dense colonies with an entire margine, and bigger in size than *Lactobacillus* colonies. *Lactobacillus* colonies were white translucent circular raised. They were smaller than *Bifidobacterium* colonies.

Two colonies from control plate of Rogosa S/L agar plate and two colonies from control plate of MRS plate were randomly selected for gram staining. *Lactobacillus* colonies were gram positive, rod-shape bacteria which colored in blue or light purple after gram staining. *Bifidobacterium* were gram positive, often branched, rod shape bacterium which colored blue or dark purple after gram staining.

**Genotype identification**

The ZR Fungal/Bacteria DNA MiniPrepTM kit (Catalogue Number: D6005, Zymo Research, Irvin, CA, USA) was used to extract ultrapure DNA from samples from isolated colonies cultured on broth media (Difco, USA). The 16s rDNA primers were selected from previous studies, retrieved from the Basic Local Alignment Search Tool (BLAST) database (Table 1), and synthesized by First Base Laboratories Sdn. Bhd. (Selangor, Malaysia).

##### Table 1: Characteristics of primers

| Target | Primer | Sequence (5’🡪3’) | (°C)  Tm | BLAST ID number | bp |
| --- | --- | --- | --- | --- | --- |
| *Lactobacillus* spp. [1] | F_alllact_IS R_alllact_IS | TGG ATG CCT TGG CAC TAG GA  AAA TCT CCG GAT CAA AGC TTA CTT AT | 58  58 | 1024485925-024664-30598 1024478788-024701-16287 | 92 |
| *Biﬁdobacterium* spp. [2] | F_allbif_IS  R_allbif_IS | GGG ATG CTG GTG TGG AAG AGA  TGC TCG CGT CCA CTA TCC AGT | 60  57 | 1015399960-19603-31240  1015400076-20827-17418 | 231 |

BLAST, Basic Local Alignment Search Tool; bp, base pair; ID, identification number; Tm, melting temperature; spp, subspecies plural.

The amplification procedure for *Lactobacillus* spp. consisted of one cycle at 94°C for 4 minutes, 30 cycles at 94°C for 30 seconds, 1 minute at 56°C as the annealing temperature, one cycle at 72 °C for 30 seconds, and one cycle of 72°C for 1 minute [1]. The amplification procedure for *Bifidobacterium* spp. consisted of one cycle at 50°C for 2 minutes, 45 cycles at 95°C for 15 seconds, 1 minute at 56.4°C as the annealing temperature, one cycle at 72 °C for 30 seconds, and one cycle at 72°C for 1 minute [2]. The amplification products were detected using agarose gel electrophoresis and UV transillumination. The gel was visualized using an alpha imager.

After PCR procedure, PCR products were visualized. Since bp of *Lactobacillus* spp. primer was 92, the *Lactobacillus* bounds were visualized in slightly lower than 100 bp (Figure 1). Similarly, since the bp of *Bifidobacterium* spp. primer was 231 (Figure 2), the *Bifidobacterium* bound was visualized higher than 200 bp. The associated colonies were confirmed as *Lactobacillus* and *Bifidobacterium*.


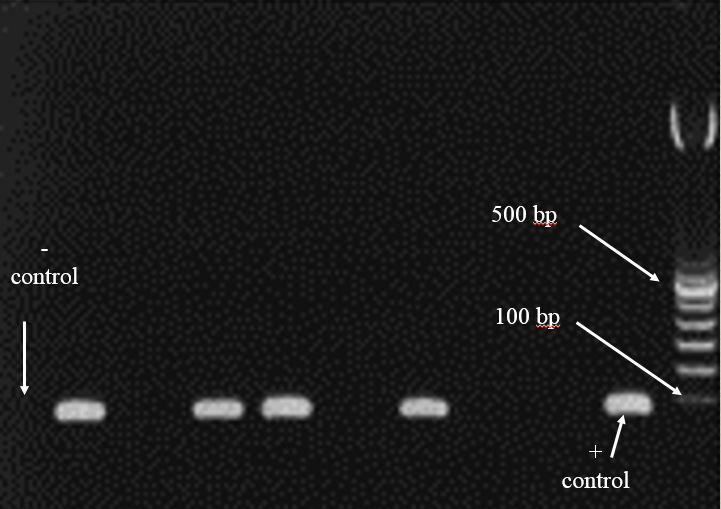


**Figure 1: *Lactobacillus* bounds obtained from PCR**


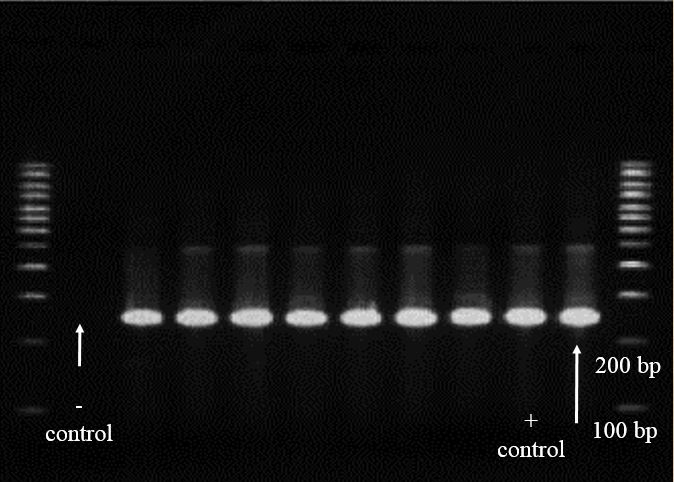


**Figure 2. *Bifidobacterium* bounds obtained from PCR**

Among the subsample of 40 subjects, four (two from each group) withdrew or dropped out of the study. Therefore, quantities of CFUs of *Lactobacillus* spp. and *Bifidobacterium* spp. were determined from 36 subjects (18 from each group).

Additional file 3 shows that quantities of *Lactobacillus* spp. increased in both groups. While this increase was not significant in Placebo Group, it increased up to two-folds in Probiotic Group. This increase was only significant in PP analysis.

While the CFUs of *Lactobacillus* spp. increased marginally (almost two-fold) in the Placebo Group, they increased significantly (up to 4.5-fold) in the Probiotic Group during both ITT and PP analysis.

**References**

1. Haarman M, Knol J. Quantitative Real-Time PCR Analysis of Fecal Lactobacillus Species in Infants Receiving a Prebiotic Infant Formula Quantitative Real-Time PCR Analysis of Fecal Lactobacillus Species in Infants Receiving a Prebiotic Infant Formula. Appl Environ Microbiol. 2006;72:2359–65.

2. Haarman M, Knol J. Quantitative Real-Time PCR Assays To Identify and Quantify Fecal Bifidobacterium Species in Infants Receiving a Prebiotic Infant Formula Quantitative Real-Time PCR Assays To Identify and Quantify Fecal Bifidobacterium Species in Infants Receiving a Prebio. Appl Environ Microbiol. 2005;71:2318–24.
